# LncRNA-uc002mbe.2 Interacting with hnRNPA2B1 Mediates AKT Deactivation and p21 Up-Regulation Induced by Trichostatin in Liver Cancer Cells

**DOI:** 10.3389/fphar.2017.00669

**Published:** 2017-09-25

**Authors:** Ting Chen, Chengxin Gu, Cailin Xue, Tao Yang, Yun Zhong, Shiming Liu, Yuqiang Nie, Hui Yang

**Affiliations:** ^1^Department of Gastroenterology, The Second Affiliated Hospital of Guangzhou Medical University Guangzhou, China; ^2^Department of Hepatobiliary Surgery, The Second Affiliated Hospital of Guangzhou Medical University Guangzhou, China; ^3^Guangzhou Institute of Cardiovascular Disease Guangzhou, China; ^4^Department of Gastroenterology, Guangzhou First People’s Hospital of Guangzhou Medical University Guangzhou, China

**Keywords:** lncRNA, TSA, hepatocellular carcinoma, hnRNPA2B1, AKT, p21

## Abstract

Long non-coding RNAs (lncRNAs) have been implicated in liver carcinogenesis. We previously showed that the induction of lncRNA-uc002mbe.2 is positively associated with the apoptotic effect of trichostatin A (TSA) in hepatocellular carcinoma (HCC) cells. The current study further analyzed the role of uc002mbe.2 in TSA-induced liver cancer cell death. The level of uc002mbe.2 was markedly increased by TSA in the cytoplasm of HCC cells. Knockdown of uc002mbe.2 prohibited TSA-induced G2/M cell cycle arrest, p21 induction, and apoptosis of Huh7 cells and reversed the TSA-mediated decrease in p-AKT. RNA pull-down and RNA-binding protein immunoprecipitation (RIP) assays revealed that TSA induced an interaction between uc002mbe.2 and heterogeneous nuclear ribonucleoprotein A2B1 (hnRNPA2B1) in Huh7 cells. This interaction mediated AKT deactivation and p21 induction in liver cancer cells. In an athymic xenograft mouse model, knockdown of uc002mbe.2 significantly prohibited the TSA-mediated reduction in tumor size and weight. In addition, the ability of TSA to reduce hnRNPA2B1 and p-AKT levels and induce p21 in the xenograft tumors was prevented by uc002mbe.2 knockdown. Therefore, the interaction of uc002mbe.2 and hnRNPA2B1 in mediating AKT deactivation and p21 induction is involved in the cytostatic effect of trichostatin in liver cancer cells.

## Introduction

Long non-coding RNAs (lncRNAs) have many functions, such as modulating gene transcription, epigenetic signaling, and protein trafficking (Evans et al., 2016; Su et al., 2016; Chandra Gupta and Nandan Tripathi, 2017). A large body of evidence has shown that lncRNAs are likely prospective novel biomarkers and therapeutic targets in cancers (George and Patel, 2015; Evans et al., 2016; Jiang et al., 2016). The functions and activities of lncRNAs depend on their subcellular distribution (Cao et al., 2015; Chen, 2016). HULC (Highly Up-regulated in Liver Cancer) is the first characterized oncogenic lncRNA in hepatocellular carcinoma (HCC). HULC exerts its oncogenic effects by promoting cell proliferation, migration, and invasion. In addition, it attenuates the sensitivity of HCC cells to chemotherapeutic agents (Panzitt et al., 2007; Li et al., 2017; Xiong et al., 2017). A lncRNA activated by TGF-β (lncRNA-ATB) promotes the invasion-metastasis cascade and is associated with poor prognosis of HCC (Yuan et al., 2014). Our published data revealed that lncRNA-uc002mbe.2 levels are reduced in HCC cell lines and liver cancer tissues (Yang et al., 2013). Furthermore, the lncRNAs RP11-134G8.8, RP11-363E7.4 and RP1-193H18.2 regulate the p53 signaling pathway and play an important role in cisplatin-induced HCC cell cycle arrest (Wang et al., 2016). Recently, microarrays were used to study the functional implications of lncRNAs in oxaliplatin-resistant HCC cells, and a series of de novo lncRNAs, including ENST00000438347, NR_073453 and ENST00000502804, were found to play important roles in HCC oxaliplatin resistance (Yin et al., 2017). Using gene expression profiling, lncRNA-Xist (X-inactive specific transcript) was identified as a biomarker that predicts the response of breast cancer cell lines (BCLs) to histone deacetylase inhibitors (HDACi) (Salvador et al., 2013). Furthermore, this previous study demonstrated that low Xist expression predicts the response to HDACi in patient-derived xenografts and is associated with a significant reduction of the breast cancer stem cells (Salvador et al., 2013). Thus, lncRNAs are novel therapeutic targets that mediate the antitumor effects of drugs such as HDACi.

Several studies have shown that increased hnRNPA2B1 expression in HCC patients is significantly associated with a poorly differentiated tumor stage and is an independent prognostic factor for HCC patients (Cui et al., 2010; Mizuno et al., 2012). HnRNPA2B1 mRNA levels are constant, and hnRNPA2B1 is thought to be a ubiquitously expressed RNA-binding protein; thus, its expression and function are dependent on posttranslational modification (He et al., 2005; Villarroya-Beltri et al., 2013).

HDACi are cytostatic agents used to combat cancer (Bots and Johnstone, 2009; Li and Seto, 2016). Previous studies by our group and others have shown that HDACi, such as TSA, have a marked ability to induce the apoptosis of liver cancer cells. Additionally, HDACi have added benefits when used in combination with synthetic retinoids, i.e., fenretinide (Yang et al., 2010; Yang et al., 2011; Li and Seto, 2016). HDACi hold enormous promise for the treatment of HCC. In our previous study, lncRNA-uc002mbe.2 underwent the greatest change among the differentially expressed lncRNAs in HCC cells after TSA exposure. Moreover, TSA-induced uc002mbe.2 levels were positively correlated with the apoptotic effects in human liver cancer cells (Yang et al., 2013). However, the underlying mechanism is not clear.

The goal of this current study was to elucidate the mechanisms by which uc002mbe.2 mediates the cytostatic effect of TSA in liver cancer cells. Our data are the first to show that TSA-induced uc002mbe.2 deactivates AKT and increases p21 by interacting with hnRNPA2B1. Additionally, the induction of uc002mbe.2 has a cytostatic effect in cancer cells and in xenograft mouse models.

## Materials and Methods

### Reagents and Cell Culture

All reagents and chemicals were from Sigma-Aldrich (St. Louis, MO, United States) unless otherwise noted. Trizol, NP40 Cell Lysis Buffer and Lipofectamine^TM^ RNAiMAX transfection reagent were purchased from Invitrogen (Carlsbad, CA, United States). Prime Script RT Reagent Kit and SYBR Premix Ex Taq were purchased from TaKaRa (Dalian, China). Annexin V-APC/7-AAD Apoptosis Detection Kit was purchased from MultiSciences (Hangzhou, China). BD Cycletest Plus DNA Reagent Kit was purchased from BD Biosciences (San Jose, CA, United States). The lncRNA FISH Detection Kit and Cell-Light^TM^ EdU Apollo^®^567 In Vitro Imaging Kit were purchased from RiboBio Co. (Guangzhou, China). Mouse monoclonal antibody against glyceraldehyde-3-phosphate dehydrogenase (GAPDH) and rabbit polyclonal antibodies against hnRNPA2B1, IGF2BP1, hnRNPU and hnRNPK were purchased from Abcam (Cambridge, MA, United States). Rabbit polyclonal antibodies specific for p-ERK, ERK, p-AKT, AKT, p-mTOR, mTOR, PTEN, p21, β-actin and cdc25C were purchased from Cell Signaling (Beverly, MA, United States). Protease and phosphatase inhibitors were purchased from Roche Applied Science (Indianapolis, IN, United States). TSA was dissolved in DMSO at 1 mM as the stock solution and stored at -20°C. The Huh7 human liver cancer cell line was purchased from Cell Cook (Guangzhou, China). Huh7 cell line was authenticated by DNA profiling via short tandem repeat analysis. Huh7 cells were cultured in Dulbecco’s Modified Eagle’s Medium (Mediatech, Herndon, VA, United States) supplemented with 10% charcoal-stripped fetal bovine serum (FBS) (Atlanta Biologicals, Lawrenceville, GA, United States) and 1% penicillin/streptomycin (Invitrogen, Carlsbad, CA, United States). The cells were cultured with DMSO, TSA (1 μM), IGF-1 (100 nM) or MG132 (2.5 μM) in media. For combination treatments, Huh7 cells were treated with IGF-1 or MG132 for 2 h before adding TSA. The final concentration of DMSO in the culture medium was 0.1% for all treatments.

### lncRNA Fluorescence *In Situ* Hybridization

The expression and localization of uc002mbe were determined by lncRNA FISH in Huh7 cells treated for 24 h with TSA according to the instructions of the Fluorescent In Situ Hybridization Kit (RiboBio, Guangzhou, China). After formaldehyde fixation, the cells were prehybridized for 30 min at 37°C and then hybridized for 12 h at 37°C with a 1:100 dilution of lncRNA FISH Probe Mix provided by the kit. After washing, the cells were stained with DAPI for 10 min and imaged by laser scanning using a confocal microscope (Carl Zeiss Company, Germany).

### LV1-shRNA uc002mbe.2 Construct and Lentiviral Transduction

LV1-shRNA uc002mbe.2 and control shGFP were purchased from TELEBIO Company (Shanghai, China). Lentiviral and packaging vectors were transfected into 293T cells. The medium was changed 8 h after transfection, and the lentivirus was collected from the medium after 48 h. Huh7 cells were infected with lentivirus in the presence of 5 μg/ml polybrene. Huh7 cells were harvested 48 h post-transfection to evaluate the efficiency of uc002mbe.2 lncRNA knockdown by quantitative real-time PCR.

### RNA Extraction and Quantitative Real-Time PCR

Total RNA was isolated using Trizol and treated with DNase I (Invitrogen, Carlsbad, CA, United States). Briefly, lncRNA levels were quantified using the Prime Script RT Reagent Kit (TaKaRa, Dalian, China) and SYBR Premix Ex Taq (TaKaRa, Dalian, China). Real-time PCR was conducted using the ABI Prism 7300 Real-time PCR System (Applied Biosystems, Foster City, CA, United States). Relative quantification was performed using the comparative CT method. The primers are listed in **Table 1**.

**Table 1:** Oligonucleotide sequences of the quantitative real-time RT-PCR or RT-PCR Primers.

Primer name	Sequence (5′– > 3′)
GAPDH	F:5-CTTTGGTATCGTGGAAGGACTC-3 R:5-CAGTAGAGGCAGGGATGATGTT-3
uc002mbe.2	F:5-TTGTCTCCCTGTTACACTGTGA-3 R:5-GGTTTATTCTTTGATGCCTTTAT-3
U1(for RIP)	F:5-GGGAGATACCATGATCACGAAGGT-3 R:5-CCACAA ATTATGCAGTCGAGTTTCCC-3
uc002mbe.2	F:5-GCTTGGGAGGAGGAGAATGCTATTTATTTCAGCACC-3
(for RIP)	R:5-CTTTGGGAGGCTGGAGGAGCTGTCCAGGG-3

### Flow Cytometric Analysis of Cell Cycle and Apoptosis

Huh7 cells were transfected with LV1-shRNA uc002mbe.2 or control shGFP for 48 h and then treated with TSA (1 μM) for 24 h. Then, cells were stained with propidium iodide using the BD Cycletest Plus DNA Reagent Kit. The relative ratio of cells in G0/G1, S, or G2/M phase was calculated. Apoptosis was evaluated using an Annexin V-APC/7-AAD Apoptosis Detection Kit. After double staining with Annexin V-APC and 7-AAD, the stained cells were analyzed using a Beckman FC 500 MOL flow cytometer with CXP LMD Acquisition and Analysis software.

### Western Blotting and Antibodies

Cells were lysed with NP40 Cell Lysis Buffer (Invitrogen, Carlsbad, CA, United States) including protease and phosphatase inhibitors (Roche Applied Science, Indianapolis, IN, United States). Equal amounts of lysates (50 μg of total protein) were separated by SDS-PAGE and transferred to PVDF membranes (Bio-Rad, Hercules, CA, United States). The membranes were blocked in PBS supplemented with 0.1% Tween 20 and 5% non-fat dry milk (PBST-milk) for 1 h at room temperature. Immunostaining was performed by incubating the membranes with primary antibodies against hnRNPA2B1, IGF2BP1, hnRNPU, hnRNPK, p-ERK, ERK, p-AKT (Thr308), AKT, p-mTOR, mTOR, PTEN, p21, β-actin, cdc25C and GAPDH in PBST-milk overnight at 4°C. After three washes, the membranes were incubated with the appropriate secondary antibody for 1 h in PBST-milk. The signal was detected using SuperSignal West Pico Chemiluminescent Substrate (Pierce, Rockford, IL, United States).

### RNA Pull-Down Assay and RNA Immunoprecipitation (RIP)

RNA pull-down assays were performed as previously described (Tsai et al., 2010; Liu et al., 2015). In brief, biotin-labeled uc002mbe.2 RNA was incubated with cellular protein extracts (1 mg), mixed with prewashed streptavidin-agarose beads for 1 h, and then washed. The uc002mbe.2-associated proteins were resolved by SDS-PAGE, and the selected bands were sent to Sai Cheng Biological Technology Co (Guangzhou, China) for identification by mass spectrometry and Western blotting. We performed RIP experiments using a Magna RIP^TM^ RNA-Binding Protein Immunoprecipitation Kit (Millipore, United States) according to the manufacturer’s instructions. The hnRNPA2B1 antibody and positive control antibody snRNP70 were used for immunoprecipitation. The RT-PCR primers are listed in **Table 1**.

### siRNA Transfections and Proliferation Assays

Scramble siRNA and predesigned siRNA specific for human hnRNPA2B1 were purchased from Santa Cruz Biotechnology (Santa Cruz, CA, United States). Huh7 cells were transfected with siRNA according to the manufacturer’s instructions. Western blotting was used to evaluate hnRNPA2B1 knockdown efficiency. The Cell-Light^TM^ EdU Apollo^®^567 In Vitro Imaging Kit (RiboBio, Guangzhou, China) was used to evaluate cell proliferation. Images were obtained and analyzed using the EVOS FL High Content Imaging System (Invitrogen, Carlsbad, CA, United States). The number of EdU-positive cells was counted in five random fields under a fluorescence microscope. The fraction (%) of EdU-positive cells was calculated as (EdU-positive cells/Hoechst-stained cells) × 400.

### Animal Experiment

Female BALB/c nude mice (5–6 weeks old, Guangdong Animal Center) were subcutaneously injected in the posterior flank on one side with 1 × 10^7^ Huh7 cells. Tumor size was measured, and tumor volume calculated using the following formula: Volume = W (width)^2^× L (length)/2. When the average tumor size reached approximately 50 mm^3^, the mice were randomly divided into two groups that received TSA (1 mg/kg/day, i.p.) plus adenoviral uc002mbe.2 shRNA or control (shGFP) (1 × 10^8^ pfu intratumoral injection, once a week). Tumor size was measured weekly using calipers, and all the mice were killed 14 days after initiating treatment. All experimental protocols were approved by the Animal Care and Use Committee of Guangzhou Medical University.

### Statistical Analysis

Data are expressed as the mean ± SD. Statistical analysis was performed using Student’s *t*-test or one-way ANOVA. The difference in tumor size was determined by repeated-measures analysis of variance. Significance was defined by *p* < 0.05.

## Results

### uc002mbe.2 Mediated the Cytostatic Effect of TSA in HCC Cells

**FIGURE 1:**
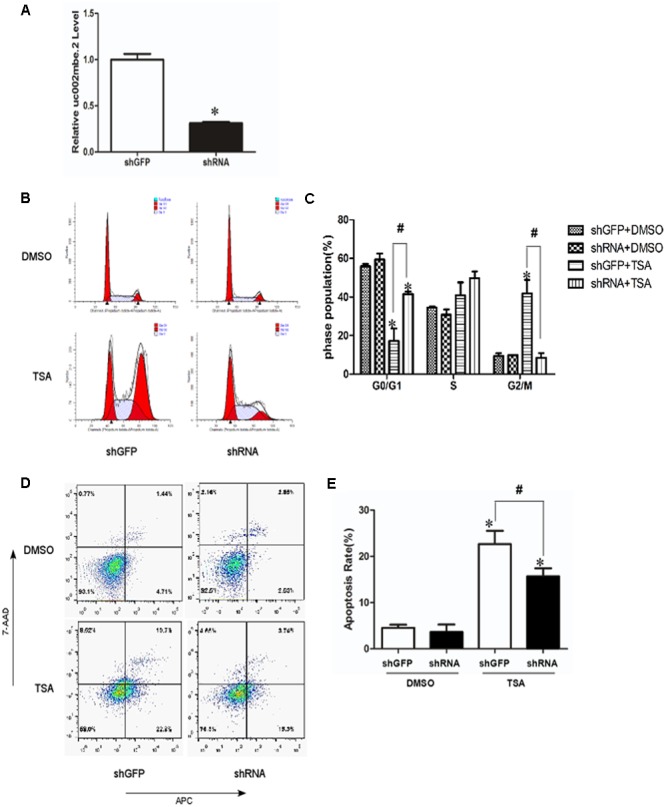
Knockdown of uc002mbe.2 inhibits the TSA-induced G2/M cell cycle arrest and apoptosis of Huh7 cells. **(A)** Huh7 cells were harvested 48 h post-transfection to evaluate the efficiency of lncRNA uc002mbe.2 knockdown by quantitative real-time PCR. **(B)** The cell cycle distribution of transfected Huh7 cells treated with either DMSO or TSA (1 μM) for 24 h was determined by fluorescence activated cell sorting. **(D)** Percentage of transfected Huh7 cells treated with either DMSO or TSA for 24 h in early apoptosis. Data are presented as the mean ± SD of three independent experiments **(C,E)**. ^#^*p* < 0.05 and ^∗^*p* < 0.05 vs. shRNA DMSO or shGFP DMSO treatment group.

To investigate the role of TSA-induced uc002mbe.2 in mediating cell death, uc002mbe.2 expression was knocked down by shRNA, and the cells were analyzed using flow cytometry assays. Transfection of uc002mbe.2 shRNA evoked an approximately 80% reduction in uc002mbe.2 levels (**Figure 1A**). Flow cytometry data showed that TSA increased the number of cells in G2/M phase but reduced the number of those in G0/G1 (**Figures 1B,C**). Knockdown of uc002mbe.2 significantly blocked TSA-induced G2/M cell cycle arrest and increased the number of Huh7 cells in G0/G1 (**Figures 1B,C**). However, uc002mbe.2 knockdown alone did not affect the number of untreated Huh7 cells in G0/G1, S and G2/M phase (**Figures 1B,C**). Annexin V-APC/7-AAD staining was also performed to study the role of TSA-induced uc002mbe.2 in mediating the apoptosis of Huh7 cells. The knockdown of uc002mbe.2 significantly inhibited the induction of early apoptosis by TSA. However, in the absence of TSA, knocking down uc002mbe.2 had no effect on the apoptosis of Huh7 cells (**Figures 1D,E**). TSA-induced apoptosis was also prevented by knockdown of uc002mbe.2 in Hep3B cells, however, uc002mbe.2 knockdown alone had no effect on the apoptosis of in Hep3B cells (Supplementary Figure 1). These findings indicate that uc002mbe.2 is essential for TSA-induced G2/M cell cycle arrest and apoptosis in HCC cells.

### p21 Induction and AKT Deactivation Are Downstream of uc002mbe.2 Induction in Huh7 Cells

**FIGURE 2:**
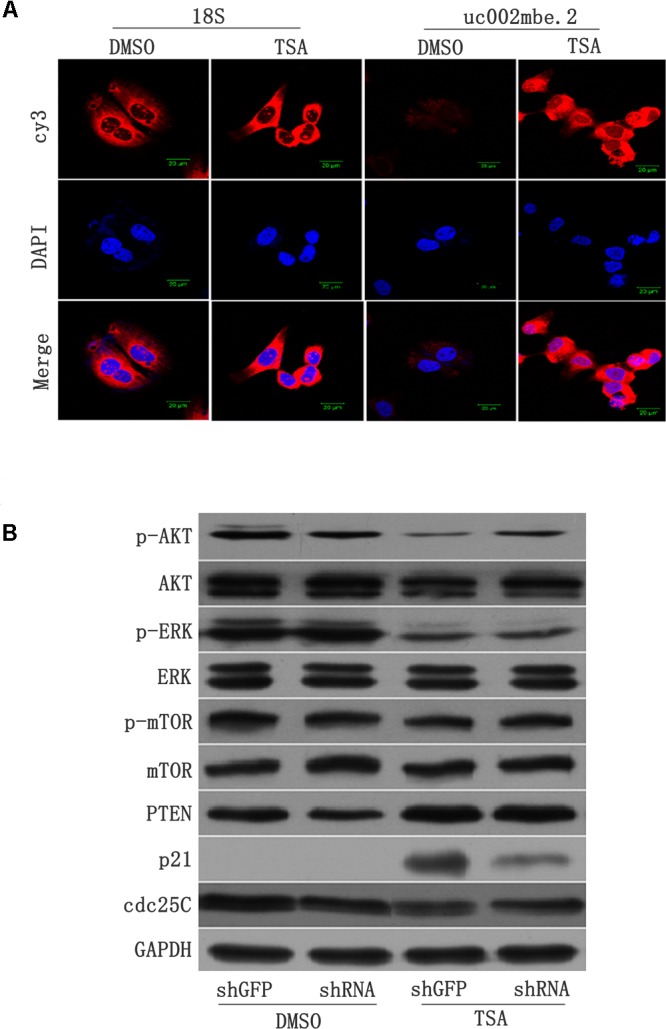
**(A)** lncRNA-uc002mbe.2 was mainly localized in the cytoplasm. Huh7 cells were treated with TSA (1 μM) or DMSO for 24 h and then subjected to lncRNA FISH assays as described in the Materials and Methods. 18S RNA was used as an internal control. The lncRNA-uc002mbe.2 and 18S RNA FISH probes were labeled with Cy3. DAPI was used as a nuclear counterstain. The images were viewed using a confocal microscope (400X). **(B)** p21 induction and AKT deactivation were downstream of uc002mbe.2 induction in Huh7 cells. Huh7 cells were transfected with either shGFP or shRNA-uc002mbe.2 for 48 h. The transfected cells were treated with TSA (1 μM) for an additional 24 h and then subjected to protein extraction for Western blot analysis using specific antibodies as described in “Materials and Methods section – Western Blotting and Antibodies”. GAPDH served as a loading control. Representative data from three independent experiments are shown.

FISH data revealed cytosolic uc002mbe.2 induction in Huh7 cells after TSA treatment (**Figure 2A**), suggesting that uc002mbe.2 may exert biological activity by interacting with other proteins. Our previous data showed that the functional interaction of co-expressed lncRNA with protein-coding genes was associated with cyclin-dependent kinase inhibitor activity and catalytic activity in TSA-induced HCC cell death (Yang et al., 2013). To analyze the interaction between uc002mbe.2 and co-expressed genes, related signaling pathway proteins were analyzed by Western blot. The levels of p-ERK1/2 and p-AKT were markedly reduced, but p21 protein levels were significantly increased after TSA treatment in Huh7 cells (**Figure 2B**). PTEN levels were modestly up-regulated, whereas cdc25C protein expression was modestly decreased after TSA treatment in Huh7 cells (**Figure 2B**). TSA treatment did not affect the expression of AKT, ERK, p-mTOR and mTOR in Huh7 cells. Knocking down uc002mbe.2 alone did not affect the levels of AKT, ERK, p-ERK, p-mTOR, PTEN or mTOR in untreated Huh7 cells (**Figure 2B**) but did significantly inhibit the induction of p21 and the decrease in p-AKT levels in TSA-treated Huh7 cells (**Figure 2B**). These results indicate that p21 induction and AKT deactivation are downstream of uc002mbe.2 induction by TSA in Huh7 cells.

### TSA Increases the Association of uc002mbe.2 and hnRNPA2B1 in Huh7 Cells

**FIGURE 3:**
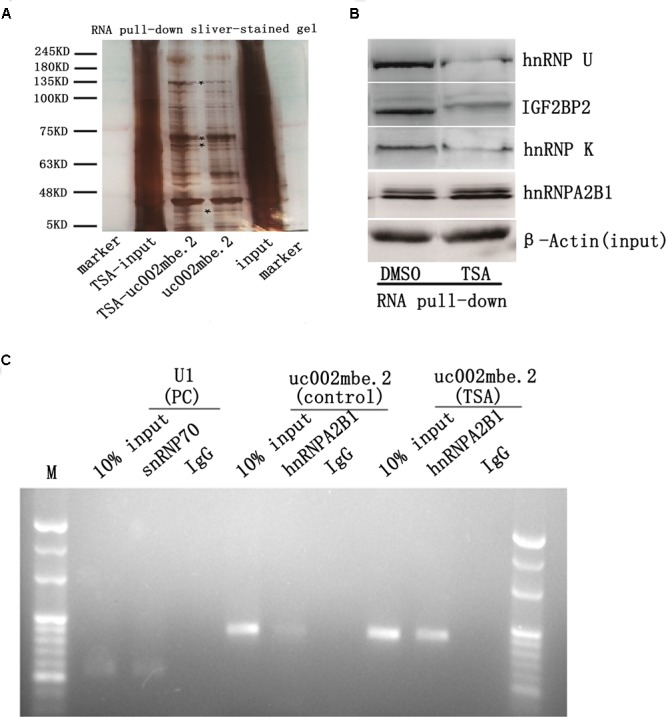
Trichostatin A increases the association of uc002mbe.2 and hnRNPA2B1 in Huh7 cells. **(A)** Proteins bound to uc002mbe.2 were resolved by SDS-PAGE. The marked regions were submitted for mass spectrometry. Identified candidate proteins are listed in **Table 1**. **(B)** TSA increased the interaction of uc002mbe.2 with hnRNPA2B1. Western blotting was performed to analyze the potential protein interactions with uc002mbe.2. β-Actin was used as an input loading control. **(C)** RIP experiments were performed in Huh7 cells after TSA treatment using hnRNPA2B1 or SNRNP70 antibodies or non-specific IgG. Purified RNA was then analyzed by RT-PCR using specific primers (listed in **Table 1**).

**FIGURE 4:**
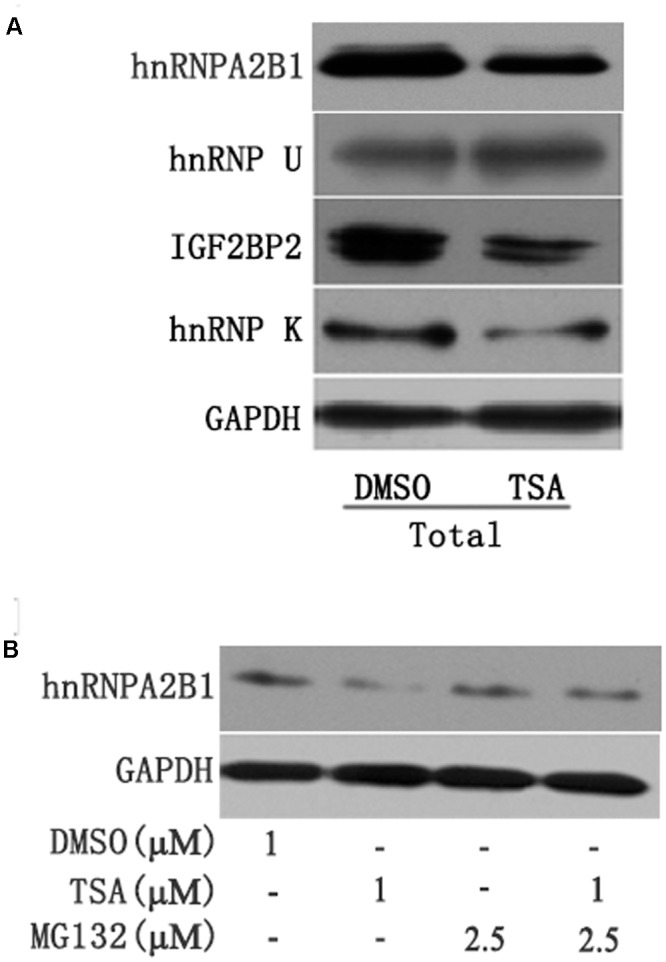
**(A)** TSA decreased the protein levels of hnRNPA2B1, IGF2BP2 and hnRNPK in Huh7 cells. Huh7 cells were treated with TSA (1 μM) for 24 h, and total protein was extracted for Western blotting using hnRNPA2B1, IGF2BP2, hnRNPK and hnRNPU antibodies. **(B)** TSA decreased hnRNPA2B1 protein levels in part via the ubiquitin-proteasome pathway. For the combination treatment, Huh7 cells were pre-treated with MG-132 (2.5 μM) for 2 h and then co-treated with TSA and MG-132 for an additional 24 h. Protein was extracted for Western blotting using specific antibodies using hnRNPA2B1 and GAPDH antibodies. Representative data from three independent experiments are shown.

Several recent studies have found that cytoplasmic lncRNAs are involved in molecular regulatory pathways through interactions with proteins (Cao et al., 2015; Atianand et al., 2016). Therefore, we hypothesized that cytosolic uc002mbe.2 might affect TSA-induced apoptosis in such a manner. We first performed RNA pull-down assays to identify proteins associated with uc002mbe.2. RNA-associated proteins were resolved by SDS-PAGE, and the selected bands were subjected to mass spectrometry (**Figure 3A** and **Table 2**). To validate the associations between uc002mbe.2 and proteins, the pull-down samples were subjected to immunoblotting. IGF2BP2, hnRNPA2B1, hnRNPU, and hnRNPK were detected by Western blot from three independent RNA pull-down assays. The associations of uc002mbe.2 with hnRNPU, IGF2BP2 and hnRNPK were significantly decreased by TSA (**Figure 3B**), but TSA increased the association of uc002mbe.2 and hnRNPA2B1 in Huh7 cells (**Figure 3B**). To further confirm the interaction between uc002mbe.2 and hnRNPA2B1, we performed RNA immunoprecipitation (RIP) assays using extracts from treated HCC cells and an antibody against hnRNPA2B1. The amount of uc002mbe.2 RNA in the co-precipitate was measured by RT-PCR. The data showed significant enrichment of uc002mbe.2 in TSA-treated samples compared with control samples (**Figure 3C**). The positive control U1 snRNA PCR product was also observed in the anti-SNRNP70 RIP. The non-specific antibody IgG was used as a negative control; no uc002mbe.2 enrichment was found in either control group (**Figure 3C**). The RIP data confirmed that TSA increased the association of uc002mbe.2 and hnRNPA2B1 in Huh7 cells. Our data also showed that TSA decreased hnRNPA2B1, IGF2BP2 and hnRNPK protein levels but did not affect hnRNPU levels in Huh7 cells (**Figure 4A**). These data indicated that TSA significantly increased the interaction between uc002mbe.2 and hnRNPA2B1. Thus, we further studied the role of hnRNPA2B1. To determine whether TSA reduced hnRNPA2B1 protein stability, Huh7 cells were treated with the proteasome inhibitor MG-132 and TSA. As shown in **Figure 4B**, MG-132 abolished the TSA-induced down-regulation of hnRNPA2B1 in Huh7 cells. The present data also showed that uc002mbe.2 knockdown alone had no signifcant effect on hnRNPA2B1 expression in HCC cells (Supplementary Figure 2). In additioanl, the interaction of uc002mbe.2 with hnRNPA2B1 might be invovled in TSA-induced hnRNPA2B1 down-regulation.

**Table 2:** Identities of putative proteins targeted by uc002mbe.2.

Treatment	Identity	Score
DMSO	IGF2BP1	1048.61
	HNRNPAB	1498.68
	HNRNPD	1487.75
	HNRNPA3	1881.95
	HNRNPA2B1	1004.21
	SYNCRIP	2014.00
	A1CF	1340.61
	IGF2BP2	1048.61
	HNRNPK	1713.98
	HNRNPU	1393.74

TSA	HNRNPA3	1712.77
	HNRNPA2B1	16994.76
	HNRNPAB	1498.68
	HNRNPL	1075.61
	IGF2BP2	1871.90
	IGF2BP1	1499.71
	A1CF	1350.65
	HNRNPK	1258.57
	HNRNPU	1393.74
	PEX11A	1646.87

### Inhibition of hnRNPA2B1 Leads to AKT Deactivation and p21 Induction in Huh7 Cells

**FIGURE 5:**
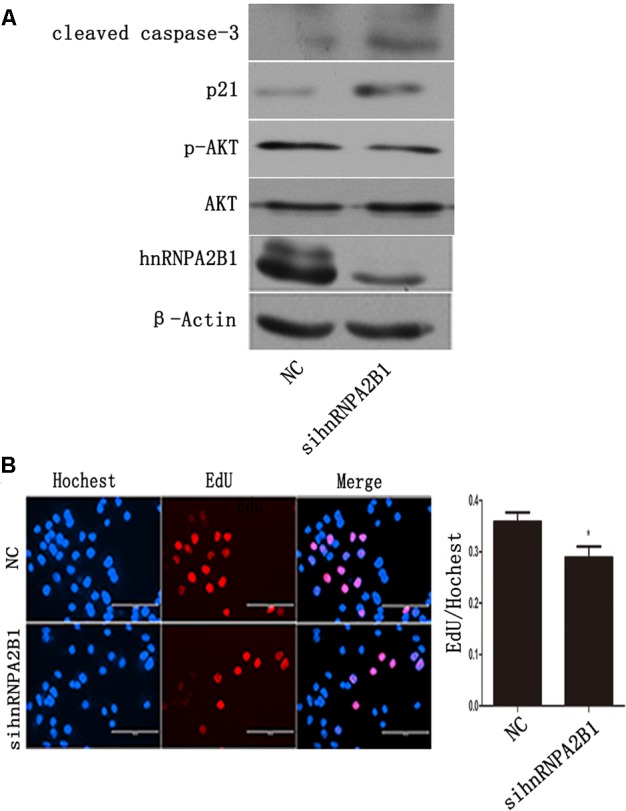
Inhibiting hnRNPA2B1 leads to AKT deactivation and p21 induction in Huh7 cells. **(A)** Huh7 cells were transfected with either si-hnRNPA2B1 or NC control for 48 h. Then, protein was extracted from transfected cells for Western blotting using specific antibodies (hnRNPA2B1, p-AKT, AKT, p21 and cleaved caspase 3). **(B)** EdU staining was used to assess proliferation after si-hnRNPA2B1 transfection in Huh7 cells. ^∗^*p* < 0.05 vs. si-hnRNPA2B1 or NC control. Representative data from three independent experiments are shown.

To assess the effects of hnRNPA2B1 in HCC cells, hnRNPA2B1 expression was knocked down using siRNA, and the cells were analyzed by Western blot and EdU staining. HnRNPA2B1 siRNA greatly reduced hnRNPA2B1 and p-AKT protein levels while increasing p21 and cleaved caspase 3 levels (**Figure 5A**). The EdU staining data showed that knocking down hnRNPA2B1 significantly reduced Huh7 cell proliferation (**Figure 5B**). These data suggested that inhibiting hnRNPA2B1 leads to AKT deactivation, p21 induction, and reduced proliferation of Huh7 cells.

### TSA-Induced p21 Expression and AKT Deactivation Are Downstream of hnRNPA2B1 Inhibition in Huh7 Cells

**FIGURE 6:**
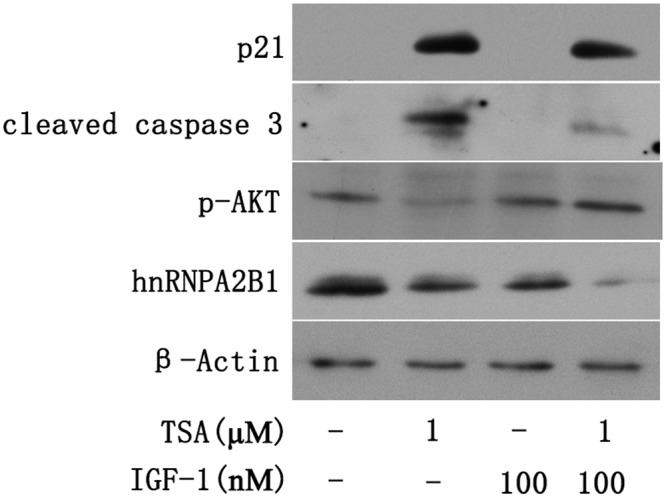
Trichostatin-induced p21 expression and AKT deactivation are downstream of hnRNPA2B1 inhibition in Huh7 cells. Cells were treated with TSA (1 μM) or IGF-1 (100 nM) for 24 h. For the combination treatment, Huh7 cells were pre-treated with IGF-1 for 2 h and then co-treated with IGF-1 and TSA for an additional 24 h. Protein was extracted from treated cells for Western blotting using specific antibodies (hnRNPA2B1, p-AKT, p21 and cleaved caspase 3).

Insulin-like growth factor-1 (IGF-1) is a mitogen that can activate AKT (Liao et al., 2016). To assess the effect of AKT on the TSA-mediated decrease in hnRNPA2B1 and induction of apoptosis, Huh7 cells were treated with IGF-1 and TSA. The apoptotic proteins cleaved caspase 3, hnRNPA2B1 and p21 were evaluated by Western blot, which showed that TSA increased cleaved caspase 3 and p21 but decreased hnRNPA2B1 and p-AKT (**Figure 6**). IGF-1 specifically activated AKT and significantly decreased TSA-induced cleaved caspase 3 in Huh7 cells (**Figure 6**). However, IGF-1 did not reverse the decrease in hnRNPA2B1 protein level and had no significant effect on p21 protein expression in Huh7 cells (**Figure 6**). The current data suggested that TSA-induced p21 expression and AKT deactivation are downstream of the inhibition of hnRNPA2B1 in Huh7 cells.

### uc002mbe.2 Interaction with hnRNPA2B1 Mediates the Antitumor Effect of TSA *In Vivo*

**FIGURE 7:**
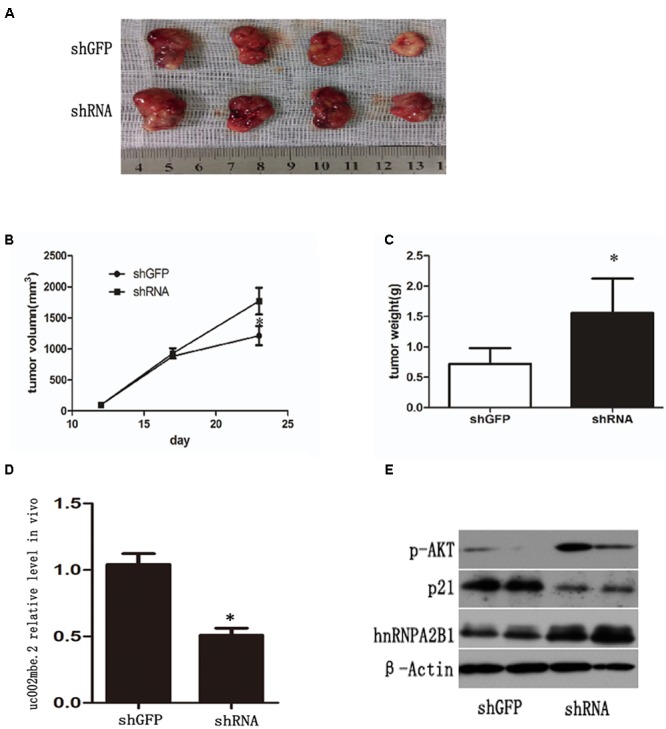
uc002mbe.2 knockdown inhibits the *in vivo* sensitivity of HCC cells to TSA. **(A)** A representative image of an isolated tumor from nude mice subcutaneously inoculated with uc002mbe.2 shRNA-transfected Huh7 cells and shGFP-transfected cells after 14 days of TSA treatment. **(B)** Tumor growth curve. **(C)** Mean tumor weight. The data are presented as the mean ± SD of 8 nude mice. ^∗^*p* < 0.05. **(D)** Total RNA extracted from isolated tumor tissue was used to evaluate the efficiency of uc002mbe.2 knockdown by quantitative real-time PCR. **(E)** Total protein was isolated from xenograft tumors and subjected to Western blotting analyses to evaluate the levels of hnRNPA2B1, p-AKT and p21.

**FIGURE 8:**
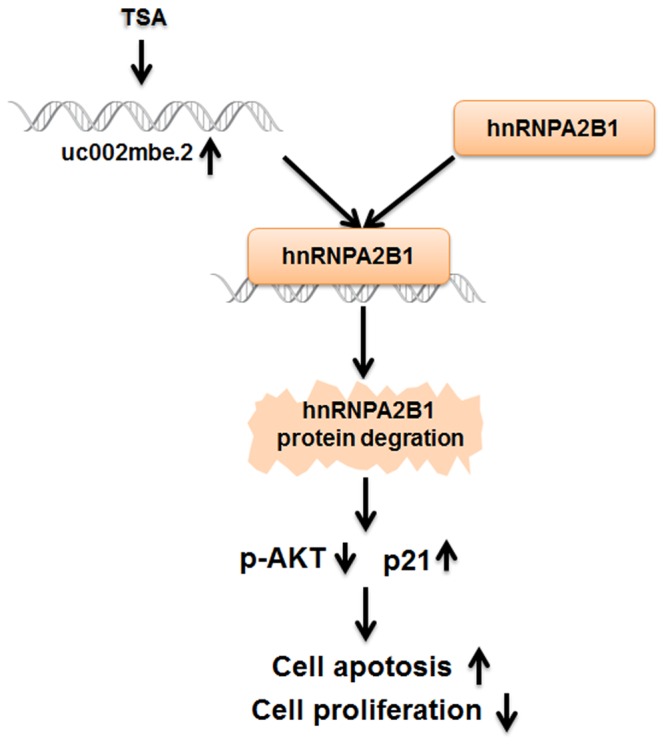
The cytostatic effect of TSA in liver cancer cells is mediated by the interaction of uc002mbe.2 and hnRNPA2B1, which leads to AKT deactivation and p21 induction. TSA-induced uc002mbe.2 directly bound to hnRNPA2B1 and promoted its degradation. The down-regulation of hnRNPA2B1 contributed to AKT deactivation and p21 up-regulation, resulting in TSA-induced cell cycle arrest and apoptosis of liver cancer cells.

A nude mouse xenograft model was used to provide further evidence for the role of uc002mbe.2 in the chemosensitivity of HCC cells to TSA *in vivo*. Tumor xenograft volume and average weight were recorded after 2 weeks of TSA treatment and intratumoral adenovirus shRNA injections. As shown in **Figures 7A–C**, uc002mbe.2 knockdown significantly inhibited the ability of TSA to reduce tumor size and weight. The qRT-PCR analysis revealed that adenovirus shRNA significantly decreased uc002mbe.2 expression in the xenograft tumors (**Figure 7D**). In addition, the ability of TSA to decrease p-AKT and hnRNPA2B1 levels and to induce p21 in xenograft tumors was prevented by uc002mbe.2 knockdown (**Figure 7E**). Taken together, our data showed that the cytostatic effect of TSA in HCC is mediated by the interaction of uc002mbe.2 and hnRNPA2B1, which leads to AKT deactivation and p21 induction *in vitro* and *in vivo*. Schematic depicting of uc002mbe.2 mediating the cytostatic effect of TSA in HCC was shown in **Figure 8**.

## Discussion

Although several lncRNAs have been implicated in chemotherapeutic sensitivity and resistance in HCC, the mechanisms underlying this drug sensitivity remain to be fully elucidated (Panzitt et al., 2007; Li et al., 2017; Xiong et al., 2017). Our data showed that knockdown uc002mbe.2 prevented TSA-induced apoptosis and G2/M cell cycle arrest in HCC cells. The level of uc002mbe.2 is substantially reduced in HCC cell lines and HCC samples (Yang et al., 2013). Due to the low basal level, uc002mbe.2 knockdown had no impact on HCC cell death. The current study is the first to provide direct evidence that by interacting with hnRNPA2B1, TSA-induced uc002mbe.2 deactivates AKT, increases p21 and has a cytostatic effect in human liver cancer cells *in vitro* and *in vivo*.

Substantial evidence indicates that lncRNAs regulate AKT signaling. High expression levels of the lncRNA Ftx and Ftx-derived miR-545 are associated with lower 5-year overall survival and disease-free survival rates of HCC patients (Liu et al., 2016). Up-regulated Ftx/miR-545 expression can induce HCC cell proliferation by activating PI3K/AKT signaling (Liu et al., 2016). HULC silencing suppresses angiogenesis by inhibiting the PI3K/AKT/mTOR signaling pathway in human gliomas (Zhu et al., 2016). Additionally, the tumor suppressing effect of lncRNAs is associated with the inhibition of the AKT signaling pathway in cancer. Down regulation of the lncRNA GAS5 is associated with a poor prognosis in prostate cancer (Xue et al., 2016). Furthermore, overexpression of GAS5 can significantly slow prostate cancer cell progression *in vitro* and tumor growth *in vivo* by inactivating the AKT signaling pathway (Xue et al., 2016). The lncRNA uc002mbe.2 deactivates AKT signaling, which plays an important role in the TSA-induced death of human liver cancer cells.

p21 is a common target of lncRNA in cancer. Focally amplified lncRNA on chromosome 1 (FAL1) promotes ovarian cancer cell growth in part by decreasing p21 expression by deregulating its transcription (Hu et al., 2014). A recent study showed that lincRNA-p21 affects global gene expression and influences the p53 tumor suppressor pathway by acting in *cis* as a locus-restricted coactivator of p53-mediated p21 expression, eventually enforcing the G1/S checkpoint (Dimitrova et al., 2014). Our data are the first to show that the lncRNA uc002mbe.2 up-regulates p21, which plays an important role in the TSA-induced cell cycle arrest of human liver cancer cells.

Recent studies showed that the cytoplasmic localization of lncRNAs enables them to interact with heterogeneous nuclear ribonucleoproteins (hnRNPs) to perform various functions (Carpenter et al., 2013; Li et al., 2014; Lan et al., 2016; Zhou et al., 2016). The interaction between cytoplasmic lincRNA-Cox2 and hnRNPA2B1 plays a key role in the transcriptional repression of target genes in the inflammatory response (Carpenter et al., 2013). lncRNA-HC was found to negatively regulate cholesterol metabolism within hepatocytes through a physical interaction with hnRNPA2B1 (Lan et al., 2016). Our RNA pull-down and RIP data showed that TSA-induced uc002mbe.2 interacted with hnRNPA2B1 in Huh7 cells. Consistent with the other findings, our data underscore the importance of hnRNPA2B1 in lncRNA function. Furthermore, data generated using the proteasome inhibitor MG-132 in combination with TSA support that TSA decreased hnRNPA2B1 protein levels in part via the ubiquitin-proteasome pathway. Additional experiments are needed to further elucidate the mechanism.

A recent study showed that phenanthrene-based tylophorine-1 (PBT-1) binds to hnRNPA2B1 and exerts antitumor activity in part by reducing AKT-mediated lung adenocarcinoma metastasis and tumorigenesis (Chen et al., 2014). The suppression of hnRNPA2B1 was associated with increased p21 and growth inhibition in HaCaT and Colo16 cells (He et al., 2005). These previous data, along with the data in the current study, indicate that the down-regulation of hnRNPA2B1 plays an important role in TSA-induced liver cancer cell death through the induction of p21 expression and deactivation of AKT.

A recent study showed that sumoylated hnRNPA2B1 controls the sorting of miRNAs into exosomes through binding to specific motifs (Villarroya-Beltri et al., 2013). Interestingly, our current data also showed that uc002mbe.2 knockdown could reverse the deregulation of hnRNPA2B1, deactivation of AKT and up-regulation of p21 induced by TSA in a xenograft mouse model. Thus, the interaction of uc002mbe.2 and hnRNPA2B1 in mediating AKT deactivation and p21 induction is involved in the cytostatic effect of trichostatin in liver cancer cells.

## Author Contributions

TC and CG: generated data, data analysis, and interpretation, manuscript preparation. CX: generated data, perform animal experiment and data analysis. TY: data analysis, and interpretation, manuscript preparation. YZ: analyzed data, generated figures and tables. SL: data analysis, and interpretation, manuscript preparation. YN: data analysis, and interpretation, manuscript preparation. HY: generated idea, study design, data analysis, and interpretation, manuscript writing.

## Conflict of Interest Statement

The authors declare that the research was conducted in the absence of any commercial or financial relationships that could be construed as a potential conflict of interest.
