# Supplementary material for: LncRNA-uc002mbe.2 Interacting with hnRNPA2B1 Mediates AKT Deactivation and p21 Up-Regulation Induced by Trichostatin in Liver Cancer Cells
Source: Front Pharmacol. 2017 Sep 25;8:669. doi: 10.3389/fphar.2017.00669 (PMC5622184; doi:10.3389/fphar.2017.00669)

**Supplementary figure 1**


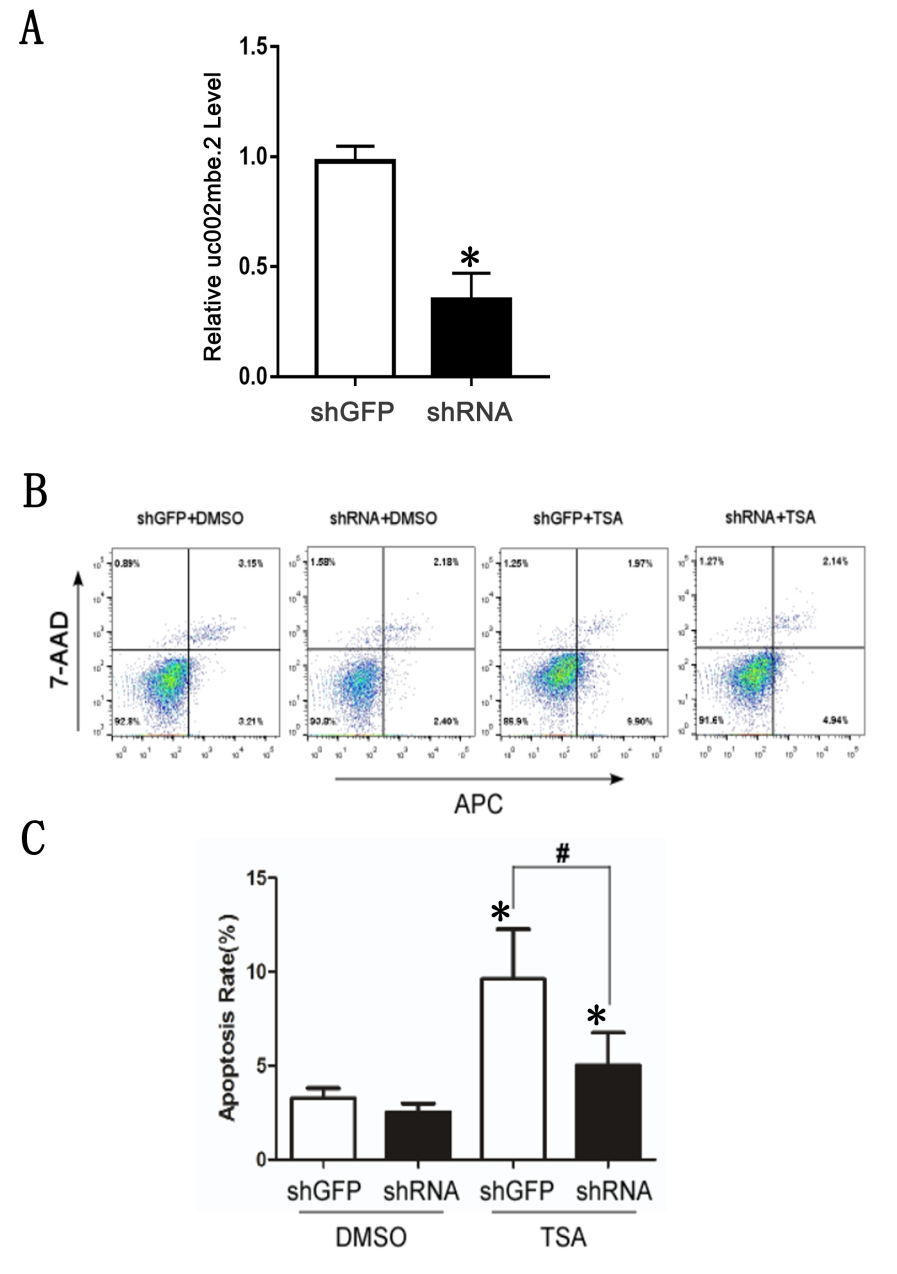


Figure 1. Knockdown of uc002mbe.2 inhibits the TSA-induced apoptosis of Hep3B cells. (A) Hep3B cells were harvested 48 h post-transfection to evaluate the efficiency of lncRNA uc002mbe.2 knockdown by quantitative real-time PCR. (B) Percentage of transfected Huh7 cells treated with either DMSO or TSA for 24 h in early apoptosis. Data are presented as the mean ± SD of three independent experiments (C). #p<0.05 and *p<0.05 vs. shRNA DMSO or shGFP DMSO treatment group.

**Supplementary figure 2**


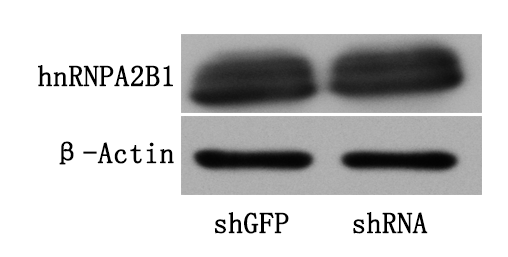


Figure 2. Huh7 were transfected with either shGFP or shRNA-uc002mbe.2 for 48 h. HCC cells were harvested 48 h post-transfection and then subjected to protein extraction for Western blot analysis using antibody hnRNPA2B1.β-Actin was used as an input loading control.


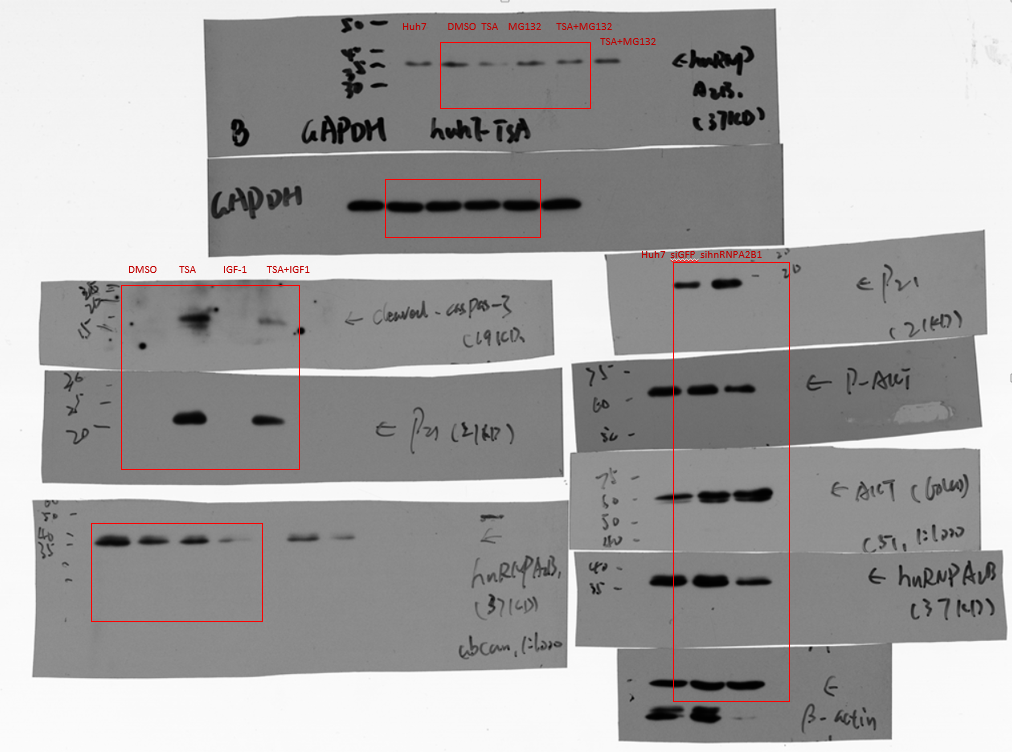

Supplement: Supplementary file 1 [file Data_Sheet_1.DOCX]
